# Association of sexual maturation with excess body weight and height in children and adolescents

**DOI:** 10.1186/1471-2431-14-72

**Published:** 2014-03-13

**Authors:** Jucemar Benedet, Adair da Silva Lopes, Fernando Adami, Patrícia de Fragas Hinnig, Francisco de Assis Guedes de Vasconcelos

**Affiliations:** 1 Programa de Pós-Graduação em Educação Física, Universidade Federal de Santa Catarina, Campus Universitário, Trindade, Santa Catarina 88040900, Florianópolis, Brazil; 2 Departamento de Educação Física da Universidade Federal de Santa Catarina, Campus Universitário, Trindade, Santa Catarina 88040900, Florianópolis, Brazil; 3 Departamento de Saúde da Coletividade, Faculdade de Medicina do ABC, Av. Príncipe de Gales, 821, Santo André, SP 09060650, Brazil; 4 Departamento de Epidemiologia da Faculdade de Saúde Pública da Universidade de São Paulo (USP), Av. Dr. Arnaldo, 715, Cerqueira César, São Paulo, SP 01246-904, Brazil; 5 Departamento de Nutrição da Universidade Federal de Santa Catarina, Campus Universitário, Trindade, Santa Catarina 88040900, Florianópolis, Brazil

**Keywords:** Obesity, Puberty, Growth, Child, Adolescent

## Abstract

**Background:**

Studies addressing the influence of early sexual maturation on the excess of body weight and height of children and adolescents are scarce. The aim of the study was to analyze the association of sexual maturation with excess body weight and height in children and adolescents.

**Methods:**

This was a cross-sectional study performed in Florianópolis city, Brazil, in 2007, with 2339 school children, aged 8–14 years (1107 males). Selection was based on a probabilistic, cluster-stratified sampling technique. School children were classified according to the presence of excess body weight, using sex- and age-specific body mass index (BMI) cutoff points. Z-scores were calculated from height and BMI data. Sexual maturation was self-assessed according to Tanner stages of development. Subjects were ranked based on tertiles of sexual maturation (early, normal and late) for each stage of development. Poisson and linear regression models were used.

**Results:**

Compared to the reference group (normal sexual maturation), early maturing females had higher prevalence of excess weight (adjusted prevalence ratio: 1.70; 95% CI: 1.24 to 2.33) and increased height-for-age (adjusted β: 0.37; 95% CI: 0.14 to 0.59), while late maturing females had lower prevalence of excess weight (adjusted prevalence ratio: 0.57; 95% CI: 0.37 to 0.87) and decreased height-for-age (adjusted β: −0.38; 95% CI: −0.56 to −0.20). In males, early and late sexual maturation were associated with increased (adjusted β: 0.37; 95% CI: 0.14 to 0.59) and decreased (adjusted β: −0.38; 95% CI: −0.56 to −0.20) height-for-age, respectively.

**Conclusion:**

Early sexual maturation is associated with excess body weight in females and with greater height-for-age in both sexes.

## Background

Excess body weight in the first two decades of life has been the focus of several studies, due to its consequences for health across the life course. This concern has been justified by the fact that excess weight prevalence is increasing in the United States [1], Europe [2] and Latin America [2,3]. The worldwide prevalence of childhood obesity increased from 4.2% to 6.7% between 1990 and 2010 [4]. In Brazil, data from nationwide surveys indicate that there was a sharp increase in the prevalence of overweight and obesity among children and adolescents between 1989 and 2009, despite a modest or no increase between 1974 and 1989 in all the country regions [5].

Puberty is a critical period for developing excess body weight, since hormonal changes impact directly on weight gain and linear growth. These changes comprise the chemical maturation of body tissues, including the amount and distribution of adipose tissue, and the increase in bone mass and in fat-free mass. In both sexes, there is a possibility of rapid increments in height and weight [6].

There are apparent differences between males and females in fat content and distribution, especially in adolescence [7]. In females, there is evidence that early sexual maturation is associated with a greater prevalence of overweight and obesity [8,9]. In males, few studies have been performed and findings are mixed [9,10].

Studies addressing the influence of early sexual maturation on the height of children and adolescents are scarce. In general, there is evidence that, regardless of the sex, those with early sexual maturation have greater height-for-age, compared to those with normal or late sexual maturation [11,12].

Thus, it is important to study the influence of sexual maturation on these anthropometric measures, given their relation to the health and development of children and adolescents. The aim of this study was to analyze the potential associations between sexual maturation and excess body weight or height in children and adolescents, aged 8 to 14 years, from the municipality of Florianopolis, in the State of Santa Catarina (SC), Brazil.

## Methods

This was a cross-sectional study performed in the city of Florianópolis, in the State of Santa Catarina, South of Brazil, during the months from April 2007 to October 2007.

The sampling procedure is described in detail elsewhere [13-15]. Briefly, for sample size calculation, the following parameters were assumed: excess weight prevalence of 22.1% for school children aged 7–10 years [16] and 12.6% for those aged 11–14 years [17]; acceptable error of 3 percentage points; two-tailed test; confidence level of 95%; design effect of 1.5; and an addition of 10% for losses. Subjects were excluded if the parents or primary caregiver did not sign the written informed consent. The sampling procedure was probabilistic, stratified by clusters and performed in two stages (school and children). Schools were grouped in four strata, according to the geographical area and school type: center/continent public schools, center/continent private schools, beaches public and beaches private schools, considering a probability proportional to the size (number of students) of each stratum. In the first stage, schools were randomly selected from each stratum. From a total of 87 schools (33 private and 54 public), 17 schools were selected (6 private and 11 public). In the second stage, school children were randomly selected according to age. For the present study, 7 year old children (n = 421) were excluded, as there was no information on sexual maturation. The final sample consisted of 2412 school children aged 8 to 14 years (1144 males, 47.4%).

Anthropometric measures were taken following the protocol of the World Health Organization [18], as proposed by Lohman [19]. Body weight was measured to the nearest of 50g using an electronic scale (model PP 180, Marte®, Minas Gerais, Brazil), capacity of 180 kg; height was measured to the nearest of 1 mm by Alturexata® stadiometer.

The team responsible for data collection was composed of 10 people previously trained in a workshop in the period of 2006 September to 2007 March. This workshop consisted of a theoretical and practical course in anthropometric measures, as well as of a pilot study in two schools (one private and one public), in which the intra- and inter- observer errors in anthropometric measures were assessed [20]. Both schools were excluded from the sampling of the study.

Sex-specific body mass index (BMI)-for-age growth charts were used to assess excess body weight, according to the World Health Organization criteria [4]. The variable excess body weight comprised the sum of overweight and obesity derived from BMI, in which 0 represents no excess of body weight and 1 represents having excess body weight. Z-scores for height-for-age, weight-for-age and BMI-for-age were also calculated for each sex separately, using LMS values. The LMS sums up the data in smoothed curves that are specific to each stratum, which in this case are the ages and genders. Parameter M is the median value of the index observed inside each stratum; parameter S is the coefficient of variation for each stratum; and parameter L is the Box-Cox coefficient employed for the mathematical transformation of the values of the variable in question in order to obtain a normal distribution in each stratum [21]. The cutoff point for biologically implausible values was 5 z-scores [4]. To calculate the z-score, LMS values were used, by age and gender, according to the following formula:

$$Z\;\text{score}\;\mathrm{BMI}=\left[{\left(\mathrm{BMI}/M\right)}^{L}‒1\right]/\left(\mathrm{LS}\right)$$

Sexual maturation was determined based on the stages of development proposed by Tanner, comprising 5 stages for genital (males) and breast (females) development [22]. School children were asked to perform a self-evaluation; the instructions were given individually.

For each sex, the age distribution (in decimals) of each sexual maturation stage was used to define tertiles of age. Based on this, individuals were classified in three groups: early sexual maturation (below first tertile of age), late sexual maturation (second tertile or more) and normal sexual maturation or reference group (between the first and the second tertile) [23]. This criterium considers that a younger age at a sexual maturation stage indicates precocious sexual maturation.

The birth weight of school children was reported by the parent or primary caregiver, who was asked to check the child’s health record. The subjects were classified into low (< 2.500 g), normal (≥ 2.500 g - 3.999 g) and high birth weight (≥ 4.000 g).

The weight status of the mother was evaluated by BMI, using self-reported weight and height. Excess body weight classification (≥ 25 kg/m^2^) followed the recommendation of the World Health Organization [24].

Information on the mode of commuting to school was collected by an illustrated questionnaire and subjects were classified as active (walking and biking) or inactive (car, bus, passenger in motorcycle or bicycle).

Further information (name, date of birth, school grade and type of school) were obtained in documents provided by the school. The type of school refers to public or private.

Given the non-normal distribution of anthropometric data (Shapiro-Wilk test, p < 0.05), quantitative variables (age, BMI Z score, height Z score, weight Z score) were described as median, 25th and 75th percentiles. Qualitative variables (excess body weight, sexual maturation classification, maternal excess body weight, birth weight, active commuting to school, school type) were described by relative frequencies (%). To compare quantitative variables between groups, Mann–Whitney (female vs. male) and Kruskal-Wallis (early, normal or late sexual maturation) tests were used. The associations among qualitative variables were assessed by Rao-Scott test. The relation between the independent variables and excess body weight (0-absence; 1-presence) was tested with univariate and multivariate Poisson regression models, using robust variance and stepwise forward strategy (multivariate model). The association between the independent variables and height was evaluated using a linear regression model (homoscedasticity checked by Breusch-Pagan and Cook-Weisberg test). Stata 11.0® was used in all statistical analysis, including the command svy to allow for sampling weights and stratification [25,26].

This study was approved by the Ethics Committee on Human Research of the Santa Catarina University (UFSC) (protocol number 028/06).

## Results

Seventy-three subjects were excluded from the analysis (loss of 3.03%) for the following reasons: height-for-age Z-score greater than 5 (1 female) and missing or inconsistent data on sexual maturation (36 males and 36 females). This resulted in a final sample of 2339 school children aged 8–14 years (1107 males). There were no statistically significant differences in excess weight prevalence based on BMI, BMI Z-score, height Z-score and the independent variables in the group excluded from the study.

Males comprised 47.7% of the study sample. When compared to females, they had higher values of BMI Z-score (p < 0.001), height Z-score (p = 0.002), greater prevalence of current excess weight (34.4 vs. 24.0%, p < 0.001) and of high birth weight (14.4 vs. 8.1%, p < 0.001). No significant differences were found in the remaining variables (Table 1).

**Table 1:** Anthropometric characteristic and mode of commuting to school in children aged 8–14 years old from Florianópolis, South of Brazil, 2007

**Study variables**	**Males (47.7%)**	**Females (52.3%)**	**p**^**§**^
	Median (p25 ; p75)*		
Age (years)	11 (10; 13)	12 (10; 13)	0.529
BMI Z score	0.51 (−0.22; 1.42)	0.21 (−0.41; 0.96)	<0.001
Height Z score	0.25 (−0.41; 0.99)	0.11 (−0.52; 0.79)	0.002
Weight Z score	0.38 (−0.20; 1.30)	0.23 (−0.55; 0.69)	0.004
	%		
Excess body weight	34.4	24.0	<0.001
Sexual maturation classification			0.937
Early	33.1	32.5	
Late	32.2	32.7	
Maternal excess body weight	31.0	33.4	0.313
Birth weight (grams)			<0.001
< 2,500	5.8	8.3	
≥ 4,000	14.4	8.1	
Active commuting to school	43.6	45.0	0.558
School type			0.975
Public	65.4	65.5	
Private	34.6	34.5	

*p25 e p75: 25th and 75th percentiles, respectively;

^§^Mann–Whitney test (quantitative variables) and Rao-Scott test (qualitative variables).

In males, sexual maturation was associated with significant differences in BMI Z-score (p < 0.001), height Z-score (p < 0.001), weight Z-score (p < 0.001) and excess weight prevalence (p = 0.027). Similar results were found in females (p < 0.001). There was also a trend towards increased BMI Z-score, height Z-score, weight Z-score and excess weight prevalence with the precocity of sexual maturation (p for trend < 0.001) (Table 2).

**Table 2:** Hypothesis tests and descriptive statistics of anthropometric variables according to sexual maturation of school children aged 8–14 years from Florianópolis, South of Brazil, 2007

	**Sexual maturation classification**					**Sexual maturation classification**				
**Variables**	**Early**	**Normal**	**Late**	**p**^**§**^	**p**^**†**^	**Early**	**Normal**	**Late**	**p**^**§**^	**p**^**†**^
	**Males**					**Females**				
	Median (p25; p75)*					Median (p25; p75)*				
BMI Z score	0.67 (−0.13; 1.56)	0.53 (−0.17; 1.41)	0.26 (−0.37; 1.11)	<0.001	<0.001	0.60 (0.01; 1.33)	0.14 (−0.40; 0.84)	−0.10 (−0.81; 0.54)	<0.001	<0.001
Height Z score	0.44 (−0.23; 1.30)	0.19 (−0.42; 0.90)	0.02 (−0.61; 0.77)	<0.001	<0.001	0.43 (−0.17; 1.07)	0.03 (−0.46; 0.57)	−0.22 (0.89; 0.61)	<0.001	<0.001
Weight Z score	0.57 (−0.09: 1.44)	0.39 (−0.19; 1.32)	0.14 (−0.38; 1.01)	<0.001	<0.001	0.46 (−0.31; 1.03)	0.24 (−0.70; 0.66)	−0.04 (−0.56; 0.49)	<0.001	<0.001
	**%**					**%**				
Excess body weight	37.5	37.8	26.9	0.027	<0.001	31.7	19.4	13.4	<0.001	<0.001

*p25 e p75: 25th and 75th percentiles, respectively;

^§^Mann–Whitney test (quantitative variables) and Rao-Scott test (qualitative variables).

^†^p for trend.

In both crude and adjusted models (Tables 3 and 4), compared to the reference group (normal sexual maturation), early maturing females had higher prevalence of excess weight (adjusted prevalence ratio: 1.70; 95% CI: 1.24 to 2.33) and increased height-for-age (adjusted β: 0.37; 95% CI: 0.14 to 0.59), while late maturing females had lower prevalence of excess weight (adjusted prevalence ratio: 0.57; 95% CI: 0.37 to 0.87) and decreased height-for-age (adjusted β: −0.38; 95% CI: −0.56 to −0.20).

**Table 3:** Estimated prevalence ratios from uni- and multivariate Poisson regression models for the prediction of excess body weight according to the sexual maturation of school children aged 8–14 years from Florianópolis, South of Brazil, 2007

**Sexual maturation classification**	**Males**			**Females**		
	Crude prevalence ratio (95% CI)*	Adjusted prevalence ratio (95% CI)*^§^	**p**^**§**^	Crude prevalence ratio (95% CI)*	Adjusted prevalence ratio (95% CI)*^§^	**p**^**§**^
Early	0.99 (0.75;1.32)	0.98 (0.73; 1.32)	0.891	1.63 (1.23; 2.16)	1.70 (1.24; 2.33)	0.004
Normal	1	1	-	1	1	-
Late	0.71 (0.55; 0.93)	0.78 (0.59; 1.02)	0.067	0.69 (0.51; 0.95)	0.57 (0.37; 0.87)	0.014

*95% confidence interval;

^§^Poisson regression model, adjusted for maternal BMI, active commuting, age, birth weight, school type and interaction between maternal BMI and school type.

**Table 4:** Estimated beta coefficients from uni- and multivariate Poisson regression models for the prediction of height Z score according to the sexual maturation of school children aged 8–14 years from Florianópolis, South of Brazil, 2007

**Sexual maturation classification**	**Males**			**Females**		
	Crude beta (95% CI)*	Adjusted beta (95% CI)*^§^	**p**^**§**^	Crude beta (95% CI)*	Adjusted beta (95% CI)*^§^	**p**^**§**^
Early	0.33 (0.12; 0.54)	0.46 (0.26; 0.66)	<0.001	0.33 (0.16; 0.50)	0.37 (0.14; 0.59)	0.005
Normal	1	1	-	1	1	-
Late	−0.23 (−0.50; 0.04)	−0.32 (−0.63; −0.02)	0.039	−0.27 (−0.39; −0.16)	−0.38 (−0.56; −0.20)	0.001
Variance homogeneity^†^	0.303			0.367		

*95% confidence interval;

^§^Linear regression model, adjusted for maternal BMI, active commuting, age, birth weight, school type and interaction between maternal BMI and school type.

^†^p value from Breusch-Pagan and Cook-Weisberg test.

In males, early and late sexual maturation were associated with increased (adjusted β: 0.37; 95% CI: 0.14 to 0.59) and decreased (adjusted β: −0.38; 95% CI: −0.56 to −0.20) height-for-age, respectively (Table 4). In contrast with the findings in females, there was no association of excess weight prevalence with early (p = 0.891) or late (p = 0.067) sexual maturation (reference group: normal sexual maturation) (Table 3). The assumption of homoscedasticity was held in the regression models of height (p = 0.303 for males and 0.367 for females, Table 4).

**Figure 1:**
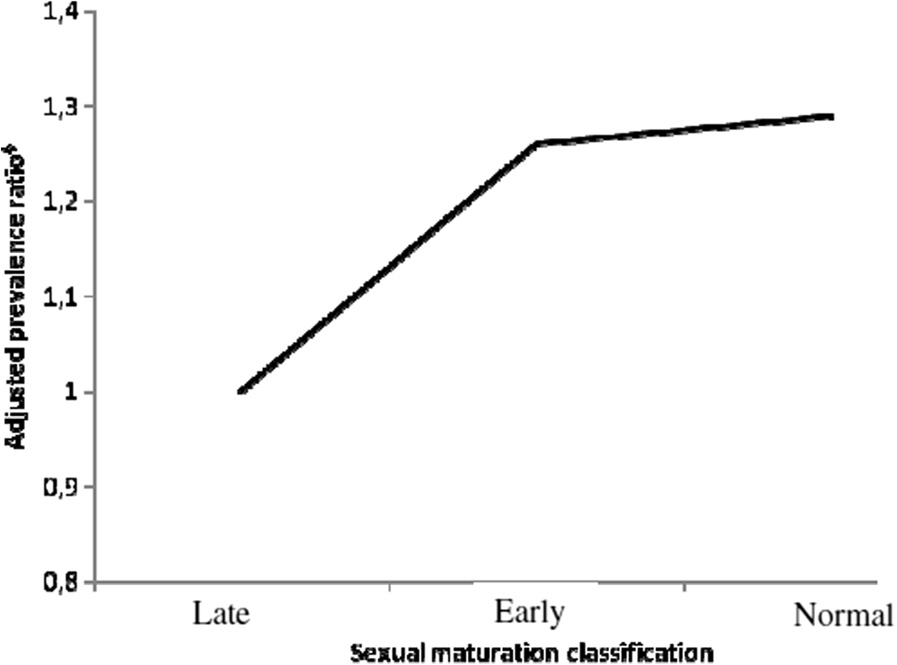
**Adjusted prevalence ratio for excess body weight by sexual maturation in male children, Brazil, 2007. **^§^Adjusted for maternal BMI, active commuting, age, birth weight, school type and interaction between maternal BMI and school type.

A trend of reduction in excess weight prevalence in males with late sexual maturation was also observed (Figure 1), despite non-significant values. Adjusted prevalence ratios of 1.26 (95% CI: 0.96 to 1.66) and 1.29 (95% CI: 0.99 to 1.67) were found in males with early and normal sexual maturation, respectively, when compared to those with late sexual maturation.

## Discussion

The study results indicate that early sexual maturation is associated with a higher prevalence of excess body weight in females and a higher stature in both sexes. The results observed in females are similar to those reported by previous cross-sectional studies [8,10,23,27].

Other cohort studies examined the association between anthropometric measures and sexual maturation in females, adjusted for BMI or adiposity in childhood [28-31]. Obesity in adulthood seemed to be more dependent on high BMI or adiposity in childhood and adolescence than on early sexual maturation [28-30]. However, such studies measured adiposity at the mean ages of 9 [29], 12 [30] and 14 [28], a period of life during which there is a potential influence of sexual maturation on body fat levels.

In contrast, studies that monitored BMI before sexual maturation showed different results. The study by Pierce and Leon [31] suggested that early sexual maturation is a stronger predictor of high BMI in adulthood than BMI in childhood. BMI was assessed between the ages 4 and 6 years, during which there is no influence of puberty in anthropometric variables. This finding indicated a relationship between early sexual maturation and obesity in adulthood, independent of BMI in childhood. Likewise, Demerath et al. [32] showed that higher BMI appears to be a consequence, not the cause, of puberty onset. The study followed females for 5 years (5 to 9 years old) and differences in weight were observed only after menarche.

The onset of puberty in females is associated with an increase in the amount of fat mass, as a consequence of increased blood concentration of estradiol [33]. Thus, it is plausible that the early onset of puberty in females is associated with a higher amount of adipose tissue, which may lead to a higher prevalence of overweight. It is noteworthy, however, that this relationship may be confounded by levels of adiposity at young ages. BMI in childhood is, therefore, an important confounding variable, given its capacity of predicting BMI in adulthood.

In males, evidence of the association between early sexual maturation and excess body weight is conflicting. In the present study, this association was not statistically significant, despite the fact that lower prevalences were observed in the group with late sexual maturation. In other studies, early sexual maturation was found to be risk factor [10], a protective factor [9] or not associated [23,28] with excess body weight. Cohort studies showed that males with early maturation have higher total adiposity [34], central adiposity [35] and waist and hip circumferences [12] in adulthood. Although it cannot be clearly stated, the body of evidence indicates that early maturing males can develop excess body weight.

Concerning height, the results showed that, regardless of sex, school children with early sexual maturation had greater height-for-age compared to those with normal or late sexual maturation. An increased height gain during childhood may be related to higher BMI and earlier puberty onset [36]. However, this greater stature is not observed in adulthood [11,12] and it seems to be restricted to late childhood and early adolescence, both in males [12,37] and in females [11]. The relationship between short stature in adulthood and early sexual maturation may be linked to the early onset of puberty and the decreased prepuberal period of growth, resulting in a premature gain in stature that does not translate into greater height in adulthood [12].

One possible explanation for the increased height due to early sexual maturation is the increased leptin levels during puberty. Studies have found that plasma leptin concentration in children and adolescents is closely related to the changes in height and body composition during growth [38]. Data from cell culture studies demonstrate that the administration of leptin stimulates the growth of cells from cartilaginous tissue [39], which can result in epiphyseal cartilage growth found at the ends of long bones. Other factors that have been implicated in the accelerated growth of obese children include increased adrenal androgens and insulin-like growth factor (IGF)-1. The highest prevalence of overweight observed among children with early sexual maturation can promote this condition because there is a positive association between levels of IGF-1 and adiposity [40,41]. In addition, it has been suggested that an increased adipose tissue aromatization of androgens into estrogens represents another important mechanism regulating growth in the context of obesity [6].

Some strengths of the study reduce the possibility of selection bias, measurement bias and confusion, such as small sample loss, the validated instruments, the trained team, and the adjustment for variables known to predict excess body weight. This, in addition to the population-based characteristic of the sample, increases the internal and external validity of the study.

The determination of early sexual maturation used in the present study, although not standardized in the scientific literature, was similar to that used by other authors [10,23]. One limitation of the study may be related to the use of self-assessed sexual maturation. However, this procedure has been validated in studies with Brazilian adolescents [42] and the results showed a good correlation (r = 0.80) between the self-assessment and experienced-personnel assessment. Data in younger populations with 8 years old or more also point to the validity of the method [43,44].

Despite the fact that BMI cannot measure body fat, it has been increasingly used to identify obese children and adolescents with high specificity, yielding few false positives [45]. Nevertheless, future studies are needed to assess the association of sexual maturity with body composition and fat distribution.

## Conclusion

Early sexual maturation is associated with excess body weight (females only) and height in school children aged 8–14 years. Undoubtedly, knowledge of the association between sexual maturation and overweight in a given population enables the implementation of preventive measures in order to reduce the exposure to these risk factors. These results are of great public health importance considering the relationship between these indicators and some noncommunicable chronic diseases, as well as the evidence that obesity in adulthood is associated with high BMI in childhood and adolescence.

## Competing interests

The authors declare that they have no competing interests.

## Authors’ contributions

All authors participated in the acquisition of data and revision of the manuscript. All authors determined the design, performed the statistical analysis, interpreted the data and drafted the manuscript. All authors read and gave final approval for the version submitted for publication.

## Pre-publication history

The pre-publication history for this paper can be accessed here:

http://www.biomedcentral.com/1471-2431/14/72/prepub
